# Metabolic Diversity and Therapeutic Potential of *Holarrhena pubescens*: An Important Ethnomedicinal Plant

**DOI:** 10.3390/biom10091341

**Published:** 2020-09-18

**Authors:** Kulsoom Zahara, Sujogya Kumar Panda, Shasank Sekhar Swain, Walter Luyten

**Affiliations:** 1 Department of Biology, Katholieke Universiteit Leuven, 3000 Leuven, Belgium; kulsoomzahara@gmail.com (K.Z.); walter.luyten@kuleuven.be (W.L.); 2 Division of Microbiology and NCDs, ICMR-Regional Medical Research Centre, Bhubaneswar 751023, Odisha, India; swain.shasanksekhar86@gmail.com

**Keywords:** ethnopharmacology, *Holarrhena pubescens*, bioactivity, phytoconstituents, pharmacokinetics, toxicity

## Abstract

*Holarrhena pubescens* is an important medicinal plant of the Apocynaceae family that is widely distributed over the Indian subcontinent. The plant is extensively used in Ayurveda and other traditional medicinal systems without obvious adverse effects. Beside notable progress in the biological and phytochemical evaluation of this plant over the past few years, comprehensive reviews of *H. pubescens* are limited in scope. It has economic importance due to the extensive use of seeds as an antidiabetic. Furthermore, the plant is extensively reported in traditional uses among the natives of Asia and Africa, while scientifical validation for various ailments has not been studied either in vitro or in vivo. This review aims to summarize information on the pharmacology, traditional uses, active constituents, safety and toxicity of *H. pubescens*. Chemical analysis of *H. pubescens* extracts revealed the presence of several bioactive compounds, such as conessine, isoconnessine, conessimine, conimine, conessidine, conkurchicine, holarrhimine, conarrhimine, mokluangin A-D and antidysentericine. Overall, this review covers the ethnopharmacology, phytochemical composition, and pharmacological potential of *H. pubescens,* with a critical discussion of its toxicity, biological activities (in vitro and in vivo), the mechanism of action, as well as suggestions for further basic and clinical research.

## 1. Introduction

Plants are long been used as a source of medicine by human beings. However, the compound(s) responsible for their therapeutic activities remained unknown for centuries. At the end of 19th century a shift occurred from natural to synthetic drugs, and phytomedicine has since progressively fallen out of use [1]. Though synthetic medicines are in use all over the world, some ancient medicinal systems still persist, e.g., Unani Tibb, Ayurveda, and traditional Chinese medicine. They make ample use of medicinal plants, which provide a source to identify chemical compounds and use them to treat different ailments [2].

*Holarrhena pubescens* Wall. ex G.Don, Syn. *Holarrhena antidysenterica* (Roth) Wall. ex A.DC. is a medicinally important plant of Africa as well as tropical and subtropical regions of Asia [3]. It is widely used in Indian medicine for treating diseases viz. diarrhea, amoebic dysentery, liver disorders, irritable bowel syndrome, and bleeding piles. The plant is astringent and bitter in taste. It is used traditionally to treat several diseases (Table 1) and there are clinical and pharmacological studies suggesting its use for various enteric, skin diseases and diabetes [4].

### 1.1. Geographical Distribution

**Figure 1:**
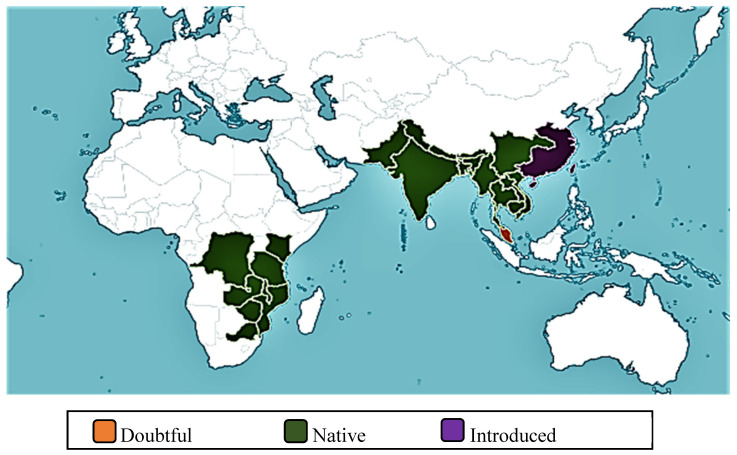
Worldwide distribution of *H. pubescens* [3].

The geographical distribution of *H. pubescens* is shown in Figure 1. It is native to South-central China, Cambodia, Myanmar, Thailand, Vietnam, India, Nepal, Bhutan, Pakistan, Bangladesh, Laos, Malawi, Mozambique, Kenya, Northern Tanzania, Zaïre, Zambia and Zimbabwe. It was introduced in South-east China, Hainan, Taiwan, and Mauritius, but its presence in Malaysia is doubtful.

### 1.2. Morphological Description

*H. pubescens* is a deciduous tree, with oblong and elliptic leaves. Flowers are white, fragrant corymbose cymes. The corolla is lobed and oblong. Fruits are slender, terete follicles, with white spots. Seeds are glabrous and linear-oblong. Its flowering season is from April–July, and fruiting is from August–October [5].

## 2. Phytoconstituents

A wide range of phytochemicals has been documented in *H. pubescens.*

- **Steroidal alkaloids**: conarrhimine, conessine, holantosines a, b, c, d, e and f, holarrhessimine, holarrhidine, holarrhine, holonamine, hydroxyconessine, kurchiline, kurchine, kurchiphylline, norconessine, n,n,n′,n′tetramethylholarrhenine, holacin, kurchinine, conamine, holadysine, 12-hydroxyconessimine, holarrhimine, holadysamine, conessimine, isoconessimine, holarosine a, conessidine, kurchiphyllamine, 7α-hydroxyconessine [26,27].
- **Uncharacterized alkaloid**: lettocine [28].
- **Triterpenes**: betulinaldehyde, ursolic acid, lupeol, 20(29)-lupadien-3β-ol, betulinic acid, lupeol β-hydroxyhexad-ecanoate [29,30].
- **Sterols**: sitosta-5,23-dien-3β-ol, stigmasterol [31].

## 3. Traditional Uses

*H. pubescens* is widely used in Ayurveda and traditional Chinese medicine. Its seeds are used as anthelminthic, and its bark is reported to have antidiarrheal properties [32]. In Ayurvedic medicine it is used for treating anemia, jaundice, dysentery, stomach pains, diarrhea, epilepsy and cholera [33]. It is widely known for the treatment of Asra (blood or blood-related disorders), Atisara (diarrhea), Kustha (leprosy), Pravahika (amebiasis), Jwaratisara (secondary diarrhea) and *Tŕṣṇā* (thirst) [34].

As described in Table 2, different parts of this plant are used by tribal communities throughout various regions of the world.

### 3.1. Bark

- In Ayurvedic medicine, its bark is used extensively for the treatment of piles, diarrhea, leprosy, biliousness and diseases of the spleen [35,36].
- In Unani medicine, bark is used to treat excessive menstrual flow, piles and headache [37].
- In British *Materia Medica,* its bark is used as antiprotozoal agent, for malaria, against chest infections, for asthma, bronchopneumonia, gastric disorders, dyspepsia, diarrhea and dysentery [38].

### 3.2. Leaf

- In Ayurveda, *H. pubescens* leaves are not reported to have medicinal value.
- In Unani medicine, leaves are used as aphrodisiac, tonic, astringent and galactagogue, and are thus used for treating chronic bronchitis, urinary discharges, wounds, ulcers, as well as for muscles relaxation; they are also useful to regulate menstruation [72].

### 3.3. Roots

Roots are reported to be aphrodisiac and abortifacient [73]. They are also used against venereal diseases, gonorrhea, ascariasis, malaria and severe abscesses [74].

### 3.4. Flowers

In Ayurveda, flowers are used as anthelmintic, antidiarrheal, and reportedly to treat leukoderma and diseases related to blood and spleen [75].

### 3.5. Seeds

**Figure 2:**
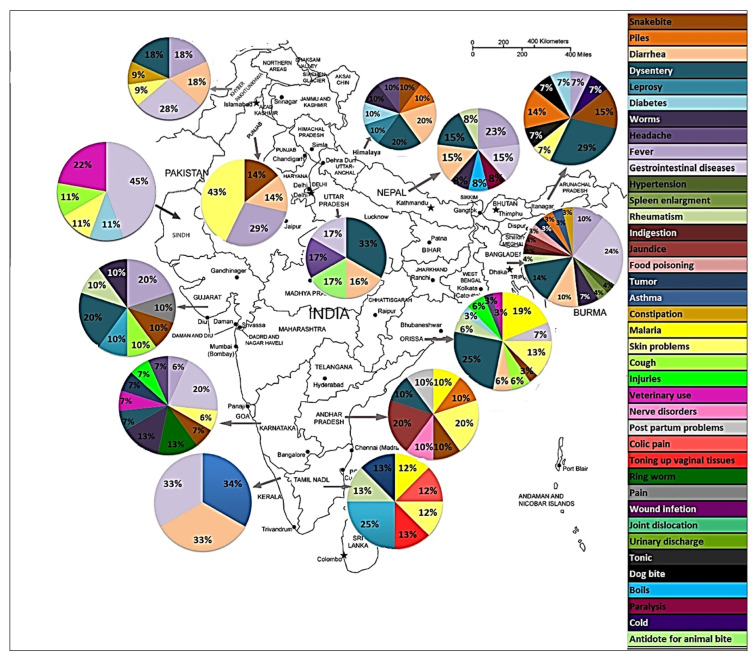
Medicinal use of *H. pubescens* in India, Pakistan, Bangladesh and Nepal.

- In Ayurveda medicine, the seeds are used as anthelmintic, astringent, and to cure dysentery, biliousness, leprosy, fatigue, skin diseases, bleeding piles, and hallucinations [76,77].
- In Unani medicine, seeds are used as carminative, aphrodisiac, astringent and lithotriptic [78].
- In Tibetan medicine, they are used as alexipharmic, antidiarrheal, cholagogue, and analgesic [79].
- In the indigenous Bangladesh system of medicine, they are used as astringent, anthelmintic, febrifuge, stomachic, anti-dysenteric and anti-diarrheal [80].
- In other parts of the world, they are reportedly used against diuresis, chronic chest infection, asthma, malaria and vaginitis [81], diabetes [82], arthritis, hematuria, epilepsy, bronchitis, diarrhea, eczema and jaundice (Figure 2).

Different *H. pubescens* parts are also used by local communities in India to treat a wide array of diseases at different dosage (Table 3).

## 4. Pharmacology

### 4.1. Anti-diabetic Property

*H. pubescens* has been used for treating diabetes in various medicinal systems. Its methanol, aqueous and petroleum ether extract of seeds are reported to have antihyperglycemic activity at a dose of 250 mg/kg body weight (BW) in rat models [108]. Keshri [109] also reported its activity against streptozotocin-induced diabetes. Especially the methanol extract of its seeds successfully protects diabetic rats at a dosage of 300 mg/kg BW (Table 4).

The ethanolic extract of its seeds significantly reduced diabetes in rats at a dose of 300 mg/kg. In a similar study on diabetic rats, decreased levels of serum cholesterol, uric acid, aspartate transaminase, triglycerides, creatinine and blood glucose were observed. Another study also demonstrated that a hydro-methanolic seed extract of *H. pubescens* causes inhibition of α glucosidase (a gut exoenzyme that releases glucose from di- and oligo-saccharides and aryl glucosides in the diet, thereby increasing absorption of glucose from the intestine) [110]. An ethanol extract of its seeds also prevented weight loss in diabetic rats and corrected biochemical parameters. With the administration of 300 mg/kg and 600 mg/kg, a significant reduction of serum cholesterol, blood glucose concentration, mean alanine aminotransferase, triglycerides, uric acid, aspartate transaminase, alanine and creatinine were reported [109].

Besides seeds, its leaves are also effective against diabetes; Hedge and Jaisal [111] reported this for an ethanolic extract of *H. pubescens* leaves at a dose of 400 mg/kg BW of rats when administrated for 21 consecutive days. The methanol extract of this plant also possesses significant (*p* < 0.05) hypoglycemic activity in vivo [112]. A study by Bhusal [113] indicated a notable antidiabetic activity, specifically with an alcoholic extract, nearly equal to standard glibenclamide.

#### Mechanism of Action

Ali et al., [10] demonstrated the effect of *H. pubescens* on α glucoside activity. α glucosidase is an enzyme which converts polysaccharides into monosaccharides. Intestines are only able to transport sugar to the blood in monosaccharide form. It was observed that an *H. pubescens* extract significantly inhibits intestinal α glucosidase with IC_50_ = 0.52 mg/mL, thus successfully limiting carbohydrate absorption. This study suggested that inhibition of α glucosidase is an important approach to limit postprandial hyperglycemia in diabetes.

### 4.2. Anti-Diarrheal Property

Diarrhea is a condition of increased secretion, volume, fluidity, and frequency of bowel movements, thus causing loss of electrolytes and water. An ethanol extract of *H. pubescens* seeds when tested on castor oil-induced diarrhea in rats is reported to cause a significant increase in the feces dry weight, and reduced defecation. At a dosage of 200 and 400 mg/kg BW, a significant reduction (*p* < 0.05) of castor oil-induced diarrhea is observed [114] (Table 4).

In another study on alkaloids isolated from *H. pubescens,* seeds were tested against clinical isolates of enteropathogenic *Escherichia coli* in vitro, and castor oil-induced diarrhea in vivo. This successfully reduced diarrhea at a dose of 200–800 mg/kg [118].

Phytochemicals i.e., saponins, steroids, alkaloids, tannins and flavonoids are reported to be responsible for the antidiarrheal activity of plants. *H. pubescens* seeds extract tests positive for alkaloids and flavonoids; therefore, these may be responsible for this activity. Aqueous and methanol extract of *H. pubescens* leaves were found effective against the diarrheal pathogens *Salmonella typhimurium, Salmonella typhi, Vibrio cholerae* and *Vibrio alginolyticus* [12].

Daswani et al. [115] tested the effect of *H. pubescens* root bark aqueous extract on *Escherichia coli*. It was observed that this plant significantly inhibits stable toxin production and reduces intestinal secretions, thus causing a decreased virulence of these enterotoxigenic *E. coli* strains. Srivastava and Saxena evaluated in vitro activity of *H. pubescens* seeds aqueous extract against diarrhea caused by bacteria like *Staphylococcus aureus, E. coli, Shigella*, and *Salmonella typhi,* and found this extract highly effective on the tested bacterial strains [119].

### 4.3. Anti-inflammatory and Analgesic Properties

*H. pubescens* extract can inhibit rat carrageenan-induced paw edema at doses of 100 and 200 mg/kg BW [116]. The methanol extract of its bark showed a decreased level of malondialdehyde and nitric oxide, but an increase in glutathione and superoxide dismutase in colitis induced in male albino Wistar rats [120]

Studies also suggest the anti-inflammatory efficacy of *H. pubescens* in a dose-dependent manner; a 400 mg/kg dose showed 74% (*p* < 0.01) inhibition when tested on carrageenan-induced rat paw edema [116]. In another study by Haque et al. [121] the methanol, petroleum ether, chloroform, dichloromethane and aqueous extract of its stem was evaluated. The chloroform extract produced maximum analgesic effect with 71% abdominal writhing inhibition, 88.5% inhibition in the open field test and CNS-depressant activity with 83% inhibition in locomotion at a 200 mg/kg dose.

In an acute inflammation model, the methanolic extract of *H. pubescens* in doses of 100 and 200 mg/kg produced dose-dependent inhibition of paw edema. The test and the standard drugs produced significant inhibition of paw edema as compared to the control (*p* < 0.001) at 3 and 4 h duration [122].

The methanolic leaf extract of *H. pubescens* (100 and 200 mg/kg) suppressed the acetic acid-induced writhing response significantly in a dose-dependent manner. In the same study, the analgesic effect was also tested in the tail flick model. Fifteen min. after drug administration, there is a significant increase in reaction time compared to the pre-drug reaction time. The extract enhances the stress tolerance capacity in animal models. The analgesic effect is proposed to be mediated by the prostaglandin pathways [123] and peritoneal mast cells [124].

### 4.4. Antioxidant/Free Radical Scavenging Properties

The aqueous and methanol extract of *H. pubescens* show a very strong radical scavenging activity with 90% DPPH free radical inhibition. The methanol extract also significantly reduces hydroxyl - and superoxide ions. In addition, it also causes a reduction of Fe^3+^ → Fe^2+^ conversion. Another study reported that its application decreases the damage to deoxyribose by OH^−^ ions. Similarly, H_2_O_2_ degradation, nitrite inhibition and lipid peroxidation were inhibited by the ethyl acetate fraction [125].

Zahin et al. [126] investigated the antioxidant capacity of *H. pubescens* by the ferric thiocyanate (FTC) method, thiobarbituric acid (TBA) method and DPPH radical scavenging method. Their results revealed a fair antioxidant effect using the FTC and TBA method, but a very low DPPH radical scavenging activity (20%). In another study conducted by Bhusal, [113] *H. pubescens* bark was evaluated for antioxidant effects, whereby both methanol and ethanol extracts had strong DPPH inhibition activity (methanol extract 96% at 0.1 mg/mL), whereas the hexane extract appeared to have the weakest activity.

### 4.5. Anti-Urolithic Property

The methanol extract of *H. pubescens* seeds was reported to have an inhibitory effect on the formation of calcium oxalate crystals. When tested in male Wistar rats, it shows a significant decrease in polyurea, Ca^++^ excretion, Ca^++^ crystal formation and water intake. These finding suggest that the plant has the potential to reduce kidney stones [117].

### 4.6. Diuretic Property

At a dose of 30–100 mg/kg in Wistar rats, the aqueous seed extract of *H. pubescens* was reported to increase urine output notably. A significant increase of excretion of Na^+^ and K^+^ ions was also observed. The chloroform extract of *H. pubescens was* also reported to cause a dose-dependent increase in urine output. In addition to this, an elevated level of urinary Na^+^ and K^+^ was also observed, thus showing that increased electrolyte excretion is probably responsible for its diuretic effect [127].

#### Gut Activities

The gut motility activity of *H. pubescens was* investigated by Gilani et al., [128]. They investigated the mechanism behind this activity of *H. pubescens* by testing extracts on high K^+^-induced contractions. The high K^+^ (> 30 mM) causes contraction of smooth muscles by opening L-type Ca^++^ channels, thus allowing entry of Ca^++^ in the cell and producing a contractile effect. Thus, an inhibitor of K^+^-induced contraction is an inhibitor of the Ca^++^ influx. In their study, hydro-ethanolic crude extract of *H. pubescens* relaxed the high K+-induced contractions just like a standard Ca++ antagonist. Thus, it was concluded that it effectively causes Ca^++^ channel blocking. Therefore, these extracts may be effective for treating gut disorders such as abdominal cramps and diarrhea.

### 4.7. Inhibition of Acetylcholinesterase and CNS-Stimulant Activity

In a study on alkaloids isolated from *H. pubescens*, five alkaloids were tested for CNS-stimulant activity i.e., conimine, isoconessimine, conessine, conarrhimine and conessimine. Conessimine showed the highest activity with an IC_50_ value of 4 μM. This study suggests that these alkaloids can be used for the treatment of neurological disorders [129]. Another study on Swiss albino mice showed that a methanolic bark extract notably decreased the grip strength and lowered locomotive activity, thus showing a depressant effect on the CNS [130].

### 4.8. Anti-Microbial Activity

Ethanol extracts of *H. pubescens* seeds showed a concentration-dependent antibacterial activity against enteropathogenic *Escherichia coli* (EPEC). The petroleum ether extract of its bark also showed inhibition of *E. coli* at a 50 μg/mL minimum inhibitory concentration. However, compared to other plants, it showed a moderate activity [131]. Studies [118] showed that adherence of the EPEC strain to INT407 cells leads to cytoplasmic membrane damage (electron microscopic studies), apoptotic bodies by the condensation of chromatin, and mitochondrial swelling and damage (fluorescence microscopy). These effects were diminished in EPEC treated with *H. pubescens* extracts.

Methanol extracts of *H. pubescens* exhibited antibiofilm activity against *V. cholerae*. Results of gene expression studies revealed that both leaf and bark extracts down-regulate aph A or aph B, the major regulator genes modulating both virulence and biofilm formation [132].

The alkaloidal fraction of *H. pubescens* showed a borderline antifungal activity, with a minimum inhibitory concentration (MIC) of 15.6 µg per disc. The methanol extract of *H. pubescens* bark showed significant antifungal potential against *Candida albicans* [133].

Conessine is the principal alkaloid demonstrated to have antibacterial activity. Till today it has not been proven whether the antimicrobial activity is due to a single alkaloid or due to a mixture of alkaloids present in this plant. Bioassay-guided purification is lacking so far, although this approach has been reported over the past 4 decades.

#### 4.8.1. Synergy and Mechanism of Action

*Acinetobacter baumannii* and *Pseudomonas aeruginosa* are important nosocomial pathogen, and treatment options are limited. Their resistance mechanisms include the production of beta-lactamases, efflux pumps, and target-site or outer membrane modifications. When tested at 250 μg/mL, the *H. pubescens* ethanol extract showed low intrinsic antibacterial activity against *Acinetobacter baumannii* and significantly enhanced the activity of the antibiotic novobiocin (concentration = 1 μg/mL, 1/8th of its MIC). Moreover, the extract at 7.8 μg/mL, confirmed resistance-modifying ability (RMA) and may be a candidate as an alternative treatment for MDR infections due to *A. baumannii* [134]. Novobiocin was chosen because of its weak antibacterial activity against Gram-negative pathogens due to an effective permeability barrier. Interestingly, when tested at different concentration using a two-fold dilution starting from 250 µg/mL, the ethanol extract enhanced the inhibitory effects of novobiocin as well as synergetic effects against all tested clinical isolates [135]. However, the authors observed no enhancement of the accumulation of ethidium bromide after treatment with the extract, suggesting that it does not act by inhibiting MDR pumps. However, it weakened the outer membrane of the pathogen as exhibited by an increase in the N-phenyl-1-naphthylamine uptake [136].

Siriyong et al. [137] investigated the efficacy of an *H. pubescens* extract and conessine as resistance-modifying agents (RMAs) on the susceptibility of *A. baumannii* to novobiocin and rifampicin. The authors observed significant synergistic activity: the fractional inhibitory concentration (FIC) index was ≤ 0.5. To investigate the mechanism of synergism, the authors used fluorescent dyes and different efflux pump inhibitors and concluded that neither the extract nor conessine act as permeabilizers [138]. The authors also noticed an increase in pyronin Y (*p* < 0.05), while there was no accumulation of ethidium bromide, suggesting the synergism was due to interference with the AdeIJK pump, but no involvement of the AdeABC pump [139].

Antibacterial activity of *H. pubescens* against *P. aeruginosa* was confirmed by several scientist using different assays. However, its mechanism is still unclear, although the principal compound with bioactivity was the alkaloid conessine.

#### 4.8.2. Conessine is Major Compound Responsible for Antimicrobial Activity

Siriyong et al. concluded that conessine in *H. pubescens* is responsible for antibacterial activity and can be useful as a combinatory therapy to restore antibiotic susceptibility in the extensively drug-resistant *A. baumannii* [137]. Siriyong et al. [138] studied the synergistic activity of conessine in combination with various antibiotics against *P. aeruginosa* PAO1 strain K767 (wild-type), K1455 (MexAB-OprM overexpressing), and K1523 (MexB deletion). An H33342 accumulation assay was used to evaluate efflux pump inhibition, while NPN uptake was used to assess membrane permeabilisation. Except for novobiocin, all other antibiotics tested such as cefotaxime, levofloxacin, tetracycline, erythromycin, and rifampicin showed synergistic activities. The authors observed that conessine might inhibit other efflux systems present in *P. aeruginosa* as indicated by synergy inhibition in the MexB deletion strain, while the inhibition of the MexAB-OprM pump was confirmed (H33342 efflux). However, membrane permeabilisation was not observed. This suggest that conessine may be applied as a novel efflux pump inhibitor to restore antibiotic activity by inhibiting efflux pump systems in *P. aeruginosa* and other Gram-negatives. Later, the authors tested a “*P. aeruginosa* strains with defined mutations that result in the overexpression of the MexAB-OprM, MexCD-OprJ and MexEF-OprN efflux pumps” as well as a mutated strain with deletion of all these pumps. The authors also studied the effects in an in vivo *Galleria mellonella* infection model. Conessine along with levofloxacin enhanced bacterial inhibition in vitro, and restored antibiotic efficacy in vivo compared to the corresponding monotherapies. The authors conclude that conessine from *H. pubescens*, enhanced the efficacy of several antibiotics, and inhibited efflux mediated MDR, without showing any toxicity in *G. mellonella* larvae [138]

### 4.9. Anti-malarial Activity

*H. pubescens* bark chloroform extracts showed significant in vitro and in vivo anti-malarial activity when tested on *Plasmodium falciparum* isolates, and when administered to Swiss mice infected with *Plasmodium falciparum* isolates, with average IC_50_ value of 5.7 μg/mL [139,140].

Nondo et al. [141] reported that ethanol and methanol extracts exhibit significant antiplasmodial activity against *Plasmodium (P.) falciparum* with an IC_50_ = 2.43 μg/mL and 2.05 μg/mL, respectively. Moreover, the fractions isolated from *H. pubescens* roots are highly active against chloroquine-resistant *P. falciparum* (K1, Dd2) and artemisinin-resistant *P. falciparum*. Another study [24], showed that the steroidal alkaloid conessine isolated from *H. pubescens* bark showed anti-plasmodial activity with an IC₅₀ value of 1.9 μg/mL.

Verma [140] tested petroleum ether and chloroform extracts of *H. pubescens* in *P. berghei*-infected mice and showed that its bark actively inhibits parasitemia. Simonsen et al. [142] also reported that a crude extract of *H. pubescens* bark has *significant vitro* anti-plasmodial activity with an IC_50_ value of 28 μg/mL against a chloroquine-susceptible strain of *P. falciparum.* The stem, root and seeds of this plant are reported to contain a large amount of steroidal alkaloid compounds i.e., conessine, kurchine, conessidine, isoconessine, conkurchicine, and holarrhimine. The chemical compound that is thought to be responsible for the antimalarial activity is conessine, isolated from its stem [142]. Verma et al. also reported that a methanol extract of *H. pubescens* inhibits *P. berghei* growth with a 43% suppression rate [140].

### 4.10. Stracture Activity Relationship (SAR) Study

**Figure 3:**
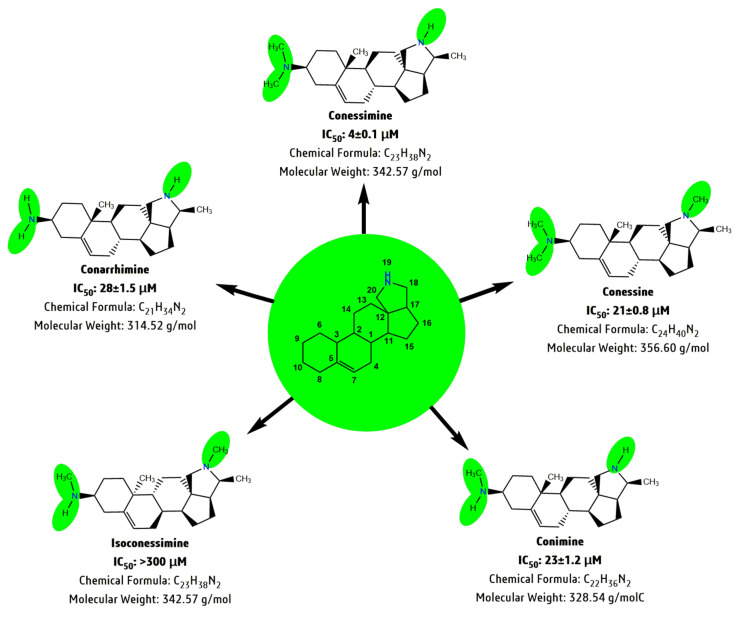
Schematic representation of the Structural-Activity-Relationship (SAR) of the steroid-alkaloid class of phytoconstituents; conarrhimine, conessimine, conessine, conimine and isoconnessine, isolated from *H. pubescens.* IC_50_ expressed in µM, range of 4 to >300 for acetylcholinesterase (AChE)/neuroprotective activity.

Currently, isolated natural products capture a lot of attention for ‘lead compound’ selection in the primary stage of drug discovery, where the SAR analysis plays a vital role in describing the structural configuration connected with biological activity and potential mechanisms of action [143,144]. For *H. pubescens*, the most common steroidal-alkaloid class of phytoconstituents; conarrhimine, conessimine, conessine, conimine and isoconnessine was reported to have multiple and dose-dependent biological activities. Structurally, the above-mentioned five compounds are derived from the “conanine” moiety: a chemical structure containing an extra pyrrolidine or tetrahydropyrrole group to the steroid moiety. For example, all five analogues showed acetylcholinesterase (AChE)/neuroprotective activity within an IC_50_ range of 4 ± 0.1 to > 300 (μM) in vitro [129]. The SAR revealed that the attachment of a methyl group (-CH_3_) to the pyrrolidine (N-atom at the position C-19 of the alkaloid moiety), as well as adding a tetradecahydro-cyclopenta-phenanthrene ring (N-atom at position C-10 of the steroid moiety), influenced AChE inhibition (Figure 3) [129]. Conessimine was the most potent AChE inhibitor, with an IC_50_ of 4 μM, where a double -CH_3_ group is present on the steroid N-atom (at position C-10), but no -CH_3_ group on the pyrrolidine N-atom (at position C-19). Similarly, the absence of -CH_3_ group at the C-19 position in both conimine and conarrhimine and the presence of a single -CH_3_ group/lack of -CH_3_ group at C-10 vary the IC_50_ between 23 to 28 μM. On the other hand, the presence of a -CH_3_ group at C-19 in conessine and double -CH3 groups at C-10 yielded an IC_50_ of 21 μM, but the presence of a single -CH_3_ group (elimination of one -CH_3_ group) at position C-10 in isoconessimine, drastically lowered the AChE inhibition (IC_50_ > 300 μM).

**Figure 4:**
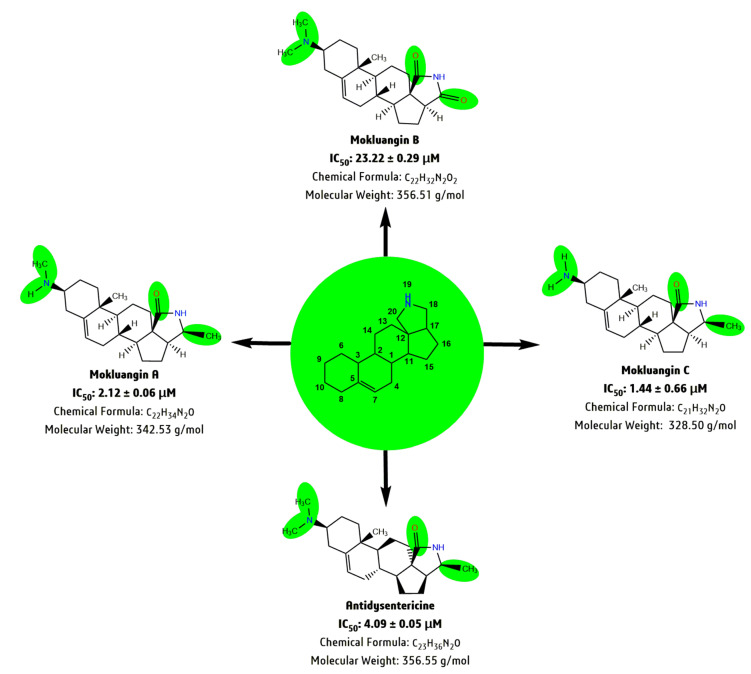
Schematic representation of the Structural-Activity-Relationship (SAR) of the steroid-alkaloid class of phytoconstituents; Mokluangin A-C and antidysentericine, isolated from *H. pubescens*. IC_50_ expressed in µM, range of 1.44 to 23.22 for acetylcholinesterase activity.

Another similar group of natural products; mokluangin A-C and antidysentericine, also exhibited AChE inhibition with an IC_50_ range of 1.44 to 23.22 μM by the presence of a carboxylic group (-C=O) at C-18 and C-20 on the pyrrolidine ring, and a -CH3 group at C-10 on the steroid moiety [145]. Regarding the SAR, the presence of a double carboxylic group at C-18 and C-20 in mokluangin B reduces AChE inhibition (IC_50_ = 23.22 μM) compared to mokluangin C (IC_50_ = 1.44 μM), mokluangin A (IC_50_ = 2.12 μM) and antidysentericine (IC_50_ = 4.09 μM), which have a -C=O at C-18 and a -CH_3_ group at C-20 (Figure 4 and [145]). Thus, in both cases, the position of the -CH3 group on the isolated natural novel steroid-alkaloid moiety plays a significant role in AChE inhibition [129,145]. Additionally, Zhao’s research demonstrated that the novel steroid-alkaloid conessine potentially crosses the brain-blood barrier at a higher rate in human and mice brain than the imidazole-containing compounds thiopermide and ciproxican, based on a cell and tissue functional assay [146].

### 4.11. Molecular Docking Studies with Conessine

**Figure 5:**
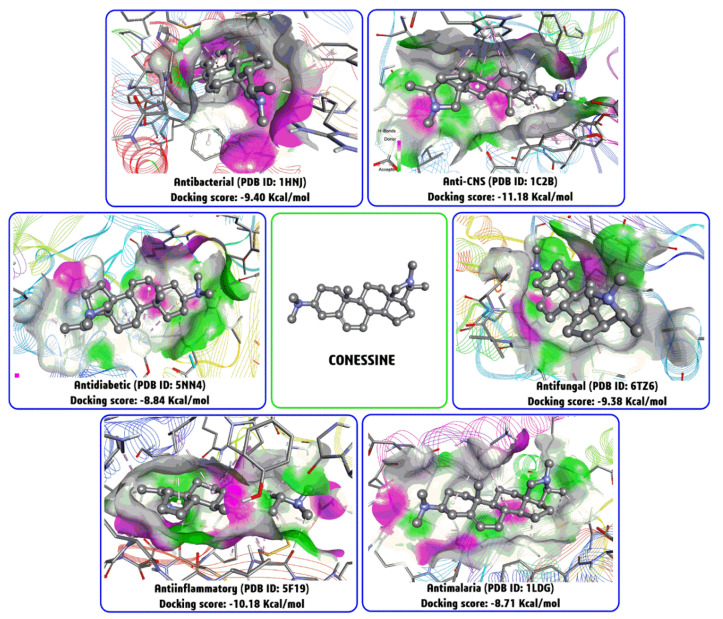
Three-dimensional molecular interaction of connesine with six different biological targets using the software, BIOVIA-DSV after a blind molecular docking study using software, AutoDock 4.2. Herein each protein data bank (PDB) ID represents the putative target proteins’ crystallographic structural information. PDB ID: 1HNJ, beta-ketoacyl-acyl carrier protein synthase III (FabH) of *E. coli*; PDB ID: 1C2B, acetylcholinesterase (AChE) of *E. electricus;* PDB ID: 5NN4, human lysosomal acid α glucosidase (GAA); PDB ID: 6TZ6, calcineurin catalytic (CnA) of *Candida albicans*; PDB ID: 5F19, human cyclooxygenase-2 (COX-2) and PDB ID: 1LDG, L-Lactate dehydrogenase (LDH) of *Plasmodium falciparum*.

Molecular docking is another artificial intelligence-based computational method for finding potential biological activities of a natural product through binding energy/docking score calculations (kcal/mol) based on “target-ligand” docking complexes [147]. Typically, the target is a macromolecule associated with the disease of interest, and the ligand is a therapeutic agent used to inhibit or activate the macromolecule and its pathways/function. A similar docking approach was also used by Cheenpracha et al., taking mokluangin A-C and antidysentericine as ligand, and the crystallographic structure of AChE reported from *Electrophorus electricus* (PDB ID: 1C2B) [145]. Similarly, using information from previous studies, six different biological activities of conessine such as antibacterial, anti-CNS, antidiabetic, antifungal, anti-inflammatory and antimalarial were analyzed through a blind docking approach [147,148]. To find out more molecular details on the binding mode of this steroid-alkaloid moiety to the presumptive molecular targets (Figure 5). Based on the individual docking score (kcal/mol), conessine would be expected to exhibited more anti-CNS activity with docking score −11.18 kcal/mol (PDB ID: 1C2B), than anti-inflammatory activity with docking score −10.18 kcal/mol (PDB ID: 1F19), antibacterial activity with docking score −9.40 kcal/mol (PDB ID: 1HNJ), antifungal activity with docking score −9.38 kcal/mol (PDB ID: 6TZ6), antidiabetic activity with docking score −8.84 kcal/mol (PDB ID: 5NN4) and antimalaria activity with docking score −8.71 kcal/mol (PDB ID: 1LDG) (Figure 5). Thus, a docking study may a cost-effective computational analysis to help understand different biological activities in the form of binding energy and possible molecular interaction-cum-mode of inhibition. Nowadays, molecular docking is also a useful tool in drug development to identify potential hit and better lead compounds, as well as insights into their mode of action [145,148].

## 5. Safety and Toxicity Studies

Various crude extracts from *H. antidysenterica* seeds such as water, ethanol, hydro alcoholic etc. were studied for their acute oral toxicity by Sheikh et al. [108] and Pathak et al. [149]; they were found to be safe up to 2000 mg/kg BW in albino rats. Singh [150], conducted a pre-clinical safety study of *H. antidysenterica* stem bark in both mice and rats. Albino mice (Swiss) treated with 2000, 1000 & 500 mg/kg, p.o. showed dullness and writhing, and a 30% mortality was recorded within 96 h. However, the authors further studied subacute toxicity in rats with lower doses (50, 100, 200 mg/kg, p.o.) and found no significant changes in hematological or biochemical parameters and histopathological examinations. An acute toxicity study conducted by Keshri et al. [109] and Kumar and Yadav [151] in albino rats revealed that ethanolic extracts of *H. antidysenterica* seeds showed no toxicity at 3000 mg/kg. Saha and Subrahmanyam [116] and Hegde and Jaisal [111] found ethanolic extracts of *H. antidysenterica* seed and leaves, respectively, to be safe when administrated at 3000 mg/kg. Other studies showed the nontoxic nature of *H. antidysenterica* when administered in albino rats at different oral doses, including 200 and 400 mg/kg [110,111,116]. A similar study conducted by Bhusal et al. [113] in male Swiss albino mice also detected no toxicity at 250 and 500 mg/kg (p.o) for methanolic extract of *H. pubescens* stembark.

In a subchronic toxicity study, an ethanol extract of *H. pubescens* along with polyvinyl pyrrolidone administrated at dosages of 270 and 530 mg/kg BW/day (which is 10 and 20 times more than the dosage used for humans), caused hepatotoxicity in rats when given for 3 consecutive months [152].

## 6. Clinical Trials

The search term “*Holarrhena*” was used to search PubMed while specifying article type “Clinical Trials”. Although no published papers with clinical studies were retrieved in this way, when we searched with the same term in Google scholar, more than a dozen publications were retrieved, most of them from India. The first clinical trial with alkaloids of *H. pubescens* for the treatment of amoebic hepatitis was carried out by Chopra and De [153]. However, they concluded that four doses of intramuscular injection of the total alkaloid mixture yields no improvement, while the patient complained of severe pain in the right hypochondriac region. Singh [154] carried out a clinical study with 40 patients suffering from intestinal amoebiasis and/or giardiasis using *H. pubescens*. In 70% of patients a response was observed in the *Entamoeba histolytica* cysts, and the authors conclude that *H. pubescens* (*Kutaja*) still remains a valuable remedy for amoebic infections.

Piles are a common chronic painful anal disease, and a clinical trial (n = 22) used a formulation containing *H. pubescens* bark (25 mg/capsule) along with six other plants [155]. All patients completed their entire study period of four weeks, and 20 out of 22 patients showed a status of wellbeing (*P*-0.01). Later, another group [156] also performed a clinical trial for piles, and observed that powder has significant role in stopping the bleeding in the disease *Shonitarsha* (bleeding piles).

A randomized controlled trial carried out by Kadam et al. [157] using a mixture of 8 herbal plants, including *H. pubescens* (1.02 g/10 g), for control of dental plaque and gingivitis. Both UDM toothpowder and standard control treatment yielded a statistically significant reduction in scores of gingival index and plaque. Another randomized clinical study with 32 children (3–12 years) with *Bhunimbadi-Vati* that contains nine herbals including *H. pubescens,* shows relief of “*Mukha Vairasya*” (bad taste in mouth) and “*Tikta Amlodgara*” (sour and better belching efficacy) [158].

The same authors carried out another randomized clinical study in 43 patients with ulcerative colitis; “*Kutaja Ghana vati*” (*H. pubescens,* 1 g three times a day) helps in reducing the bowel movement frequency [159]. Johari and Gandhi [160] carried out a randomized single-blind parallel group study comparing a monoherbal formulation containing *H. pubescens* extract with mesalamine in chronic ulcerative colitis patients, with special emphasis on side effects and relapse. The study supports the efficacy of the monoherbal formulation in resolving chronic ulcerative colitis, with fewer chances of relapse and side effects. However, the authors recommend that the study be used to conduct Phase II and III clinical trials with larger sample sizes. Recently, Kumari et al. [161] studied the efficacy of *Kutaja* syrup (*H. pubescens)* on 30 infants suffering from acute diarrhea. The trial drug was given to infants at a dose of 15 mg/kg, every 8 h for two days; the drug had a significant role in reducing signs and symptoms of diarrhea. However, the authors advised a randomized controlled trial with adequate sample size.

“*Pathadi Kwatha*” contains a mixture of five plants, including *H. pubescens;* it was tested in patients with polycystic ovarian disease (n = 34) in a randomized clinical trial, and proved statistically significantly effective in regularizing menstruation, achieving considerable reduction in body weight, substantial growth of follicles, and thus ovulation [159].

Mundhe et al. [162], performed an open-label, prospective, multi-center clinical study to evaluate the efficacy of *Ayuartis* capsules (contains *H. pubescens* stem bark 30 mg, along with several other plants) in patients suffering from osteoarthritis of the knee(s). Three months of treatment with *Ayuartis* capsule led to a significant reduction in joint pain and joint stiffness. Therefore, this can be an effective treatment option for the management of chronic degenerative joint disorders such as osteoarthritis.

In all trials where multi-herbal treatments were tested, no conclusions can be drawn about the contribution of *H. pubescens* to the therapeutic effect, if any. Even for *H. pubescens* monotherapy, the quality and size of the trials preclude definitive conclusions, and more convincing trials are needed, as many of the authors concede.

## 7. The Way Forward

*H. pubescens* has been demonstrated by different in vitro studies to have a wide range of medicinal properties, particularly anti-malarial, anti-mutagenic, anti-hypertensive, anti-diarrheal, anti-microbial, CNS-stimulant, diuretic, anti-amoebiasis, anti-urolithic, antioxidant, anti-inflammatory, gut-relaxant and anti-diabetic properties. Several bioactive chemical compounds have been isolated from this plant i.e., conimine, isoconessimine, conessine, conarrhimine and conessimine. In in vitro studies conessimine appeared to have considerable CNS-stimulant activity, as well as antibacterial effects. However, clinical trials on the therapeutic potential of this plant are limited and largely preliminary.

Therefore, it would be useful to explore the individual compounds isolated from *H. pubescens* in order to validate its ethnomedicinal uses, and to develop clinical applications of this plant. Further efforts are required to identify the active compounds using bioassay-guided purification.

## 8. Conclusions

*H. pubescens* is a well-known plant that is mostly used by indigenous communities of Asia. It is used by multiple communities for treating various ailments such as rheumatism, leprosy, skin diseases, diarrhea, dysentery, gastrointestinal infections, stomach-ache, piles, cough and cold, typhoid fever and malaria, etc. Conessine, an active compound of *H. pubescens* has demonstrated biological properties. Other major bioactive components are holarrhemine, conkurchine, kurchicine, holarrhenine, kurchine, and conkurchinine. From our brief overview, it is evident that several in vitro effects of crude extracts were reported, but in most cases further work is required to isolate and characterize the bioactive compounds. Moreover, except for the antimicrobial and acetylcholinesterase/neuroprotective activity, all other ones need further follow-up, with the mechanism of action and structure-activity relationship studies to assess more fully their potential as drug candidates.

## Figures and Tables

**Table 1:** Medicinal properties of *H. pubescens.*

Disease	Medicinal Property	Reference
Intestinal parasites	Anthelmintic for Guinea worm, roundworm, tapeworm, thread worm, other internal worms	[5]
Animal bites	Antidote for snake bite, scorpion sting, insect bite, dog bite	[6]
Indigestion	Appetizer, stomachic	[7]
Blood-related ailments	Anemia, blood infection, blood purifier, hemorrhage, nose bleeding, hypertension	[8]
Body pain	Analgesic for backache, body ache, headache, knee pain and rheumatic arthritis	[9]
Brain-related disorders	Improves depression and other nervous disorders, acts as memory enhancer	[10]
Cold and throat-related ailments	Expectorant for cold, cough, throat infection	[9,10]
Dental or oral ailments	Analgesic for toothache	[11]
Dermatological problems	Activity against abscess, acne, boils, bruises, dermatitis, leukoderma, pimples, ringworm, scabies, skin allergies, warts	[12]
Diabetes	Regulates blood sugar	[6,13]
Fever	Antipyretic, febrifuge for intermittent fever, pyrexia	[12]
Gastrointestinal disorders	Active against (hyper)acidity, intestinal ulcers, stomachache, dyspepsia, flatulence, cholera, diarrhea, dysentery, food poisoning, gastroenteritis, colic complaints, constipation	[7]
General health	Muscle strength, obesity, tonic	[14,15]
Gynecological disorders	Easy delivery, leucorrhea, toning up vaginal tissues after delivery	[16,17]
Joint- and muscle-related ailments	Active against arthritis, rheumatism	[18,19]
Liver complaints	Useful for bilious disorders, bile infection, jaundice	[20,21]
Piles	Active against piles, fissures, fistula, hemorrhoids	[22,23]
Respiratory disorders	Active against asthma, bronchitis	[24,25]
Urogenital disorders	Controls urination, cystitis, diuretic, dysuria, urinary problem, urinary tract infection, urine tract burning sensation	[25]

**Table 2:** Common traditional uses of *H. pubescens* throughout different parts of the world.

Geographic Location	Condition Treated	Plant Part Used	*Method*(s) of Preparation	*Dosage* Forms, and *Method*(s) of Administration	Reference
East Africa	Fever	Leaves, roots	Decoction	Bath is taken	[12]
	Malaria	Roots	Decoction	Taken in the form of drink twice daily	[21]
Southern Africa	Constipation, abdominal pains	Root	Infusion	Drink	[38]
	Infertility/amenorrhea	Root	Decoction	Drink	[39]
	Toothache	Stem, bark	Decoction	Gargle	[40]
	Snakebite	Root	Boiled in milk	Applied externally	
West Africa	Stomach pains	Leaves	Maceration	Drink	[40,41]
Togo Maritime region	Malaria	Leaves, roots	Decoction	Oral administration	[42]
Zimbabwe	Abortifacient/venereal diseases	Root	Infusions	Oral administration	[43]
	Malaria	Root	Decoction	Oral administration	[34,44]
Tanzania	Abdominal pain	Roots	Decoction	Taken in the form of drink on empty stomach	[45]
Mozambique	Stomachache/vomiting	Leaves, roots	Maceration	Oral administration	[46]
	Earache	All parts	Maceration	Directly applied in the form of ear drops	
Guinea	Diabetes	Whole plant	Not stated	Not stated	[13]
South West Nigeria	Inflammatory diseases	Leaves	Infusion	Oral administration	[47]
Republic of China	Diarrhea, dysentery	Bark	Decoction	Oral administration	[48]
Northern Thailand	Diarrhea and weight loss	Stem, bark	Boiled	Oral administration	[49]
India	Low fever	Seeds	Powder	Oral administration, 2–3 g mixed in one glass of water	[50,51]
	Knee pain	Bark	Decoction	Oral administration, mixed with about 100 g of jaggery	[52,53]
	Leprosy	Seeds	Decoction	Oral administration	[54,55]
	Snakebite	Roots	Paste	Directly applied to bite wound	[56]
	Dysentery	Bark, leaves	Powder	Taken with water	[57,58]
	Amoebic dysentery	Bark	Powder	Oral administration	[59,60]
Nepal	Paralysis	Bark, root	Powder	One spoonful powder or paste from a mixture of (5 g *H. pubescens* root, 5 g *Terminalia alata* bark, 2 g *Cissampelos pareira* root, 5 g *H. pubescens* bark, 2 g *Psidum guajava* bark, 1 g *Allium sativum* bulb and 2 g *Trachyspermum ammi* seeds), given once a day	[37]
	Backache, high fever	Bark	Infusion	Oral administration	[4]
Bangladesh	Bloody dysentery	Bark	Boil	1 cupful bark of *H. pubescens* is boiled with 4 cups of water to make 1 cup. A 1.5 mL solution with trace amount of honey is licked 3–4 times daily till cure	[61]
	Stomach pain, food poisoning	Bark	Maceration	A red-hot iron rod is dipped in the juice, and the juice is taken while still warm	[62]
		Bark		Mixed with bark of *Cinnamomum camphora* and chewed.	[63]
	Jaundice	Leaves	Macerated juice	Juice obtained from leaves of *Cajanus cajan* and *H. pubescens* are mixed with powdered seeds of *Plantago ovata* and taken (one glassful) in the morning on an empty stomach for one month	[64]
	Helminthiasis	Seeds	Powder	Taken with cold water every morning	[65]
	Piles	Bark	Powder	Mixed with honey and taken orally	
	Abdominal pain, diarrhea	Bark	Juice	A ½ cup is taken 2–3 times orally	[66]
	Asthma	Root	Juice	Taken 4–5 times daily for a week	
	Abdominal pain	Bark/leaf	Juice	2–3 spoons along with honey on empty stomach	
Pakistan	Diabetes	Root	Powder	*Salacia reticulate*, *Annona squamosa* and *H. pubescens* roots were Ground with lime and taken orally	[67]
	Malaria	Root	Decoction	Oral administration	[68]
	Diarrhea	Bark	Decoction	Oral administration	[69]
	Gut infections	Leaves	Juice	Taken daily	[70,71]

**Table 3:** Regional uses of *H. pubescens* by traditional healers across India.

State/Province, Tribe(s)	Disease/Indication	*Dosage* Forms, and M*ethod*(s) of Administration	References
**Tripura state**, reang tribes	Dog bite	Pills prepared from bark	[83]
Unakoti district	Antidiabetic	-	[84]
West and south district of Tripura	Dysentery, fever, cold and piles	-	[85]
**Uttar Pradesh state**	Dysentery	Bark decoction	[86]
	Diarrhea		
Sonaghati of Sonbhadra district	Stimulate discharge of urine and to remove constipation	10–20 g of root paste is taken orally with water	[32]
Jaunsar-bawar hills	Dysentery and stomachache	Dry stem bark mixed with dried ginger and black pepper are powdered and made into pills with butter oil, 2–3 of these pills (pea size) are administered daily	[75]
**West Bengal state**	Blood dysentery, piles, leprosy, headache	Bark	[87]
	Diabetes, intestinal worms; roots to stop bleeding from nose	Seeds	
	Dropsy	The dried bark is rubbed over the body	
**Madhya Pradesh state**, tribal communities of chitrakoot region	Arthritis and diarrhea in cattle	Leaf decoction twice a day	[88,89]
**Odisha state**	Rheumatism	Root bark	[90]
Tribals of Bargarh district	Rheumatism	10 g of root bark is boiled in water (400 mL) and the prepared decoction (100 mL) is taken 1–2 times daily on empty stomach	
Sundargarh district	Boils, cut, abscess and wounds	Root paste	[91]
Bondo tribe of Malkangiri district	Rheumatic pain	Two to three leaves are attached with the latex of the same plant and fomented externally over backbone	[92]
	Dysentery	Root powder	
Tribals of Similipal	Malaria and dysentery	Stem bark	[93]
	Dysentery	Stem bark infusion with honey in a ratio of 3:1 is taken once a day on empty stomach	[94]
	Dysentery	From bark of *H. pubescens, Terminalia arjuna* and *Pterocarpus marsupium* (in equal ratio) pill is prepared. One pill is taken orally on empty stomach for three days	[95]
Tribes of Mayurbhanj district	Stomach pain and blood dysentery		[94]
	Headache	Decoction of roots with garlic and mustard is made into paste and applied externally as an ointment	[95]
	Skin infection, jaundice	Leaf paste	[96]
Bhadrak district	Deep cuts	Bark and latex	[52]
Kalahandi district	Dysentery	Stem bark of *Careya arborea* and *H. pubescens* with water	[33]
**Andhra Pradesh state**, visakhapatnam district	Nerve disorder	Spoonful of shade-dried stem bark powder was taken orally with glass of water daily	[97]
Khammam district	Post-partum problems	15 g of root is ground with 20 mL country liquor of rice. Five spoons of this were taken immediately after delivery followed by 2 g of *Ferula asafoetida* rhizome powder	[98]
Visakhapatnam district	Fever	Decoction prepared by adding 100–400 mL water with leaves of *H. pubescens* and root of *Andographis paniculata,* given twice a day	[99]
**Karnataka state**, Hosanagara taluk of Shimoga district	Cancer	One handful of roots ground in cow’s buttermilk and given orally, twice daily for one month	[100]
	Stomachache	Roots crushed in water and juice is taken orally, twice daily for 1–2 days	[101]
Tribes of the Shimoga district	Ringworm and poor milk production	Bark	[102]
Uttara kannada	Ulcer in intestine	Used a mixture of plants viz. *Syzygium cumini* (bark); *Holarrhena pubescens* (bark), *Madhuca indica* (leaves and bark), *Careya arborea* (bark), *Elaegnus conferta* (bark), *Myristica fragrans* (fruit), *Syzygium aromaticum* (flower bud), *Piper nigrum* (fruit), *Trachyspermum ammi* (fruit), *Zingiber officinale* (rhizome), *Cuminum cyminum* (fruit) in decoction form	[103]
**Uttarakhand state**, Tharu community of district Udham Singh nagar	Chronic dysentery	Paste made with flower and cow’s milk taken orally, for 4 days	[104,105,106]
Theni district (Western ghats)	Dysentery	Decoction made from the root bark is taken orally twice a day for two days	[107]
**Gujrat, Rajstan** and **Kerala state**	Dropsy and swelling	Bark extracts from *Bombax ceiba, Hymenodictyon excelsium, Azadirachta indica* and *H. pubescens* made by crushing is given with water in morning and evening for 5 days	[56]
	Snakebite	The crushed root is given with ghee	

**Table 4:** In vivo studies with *H. pubescens.*

Biological Activity	Parts	Extract/Compound	Effective Concentration/Dose	Study Model	References
Antihyperglycemic	Seeds	Aqueous and petroleum ether extract	250 mg/kg BW	Rats	[109]
	Seeds	Methanol extract	300 mg/kg BW in rats	Rats	[110]
	Seeds	Ethanolic extract	300 mg/kg and 600 mg/kg	Rats	[110]
	Leaves	Ethanolic extract	400 mg/kg BW	Rats	[112]
Anti-diarrheal	Seeds	Ethanolic extract	200 and 400 mg/kg	Rats	[114]
	Seeds	Alkaloids	200–800 mg/kg	Rats	[115]
Anti-inflammatory	Not stated	Not stated	400 mg/kg	Rats	[116]
Diuretic	Seeds	Aqueous	30–100 mg/kg	Rats	[117]
